# Norepinephrine release in the cerebellum contributes to aversive learning

**DOI:** 10.1038/s41467-023-40548-8

**Published:** 2023-08-10

**Authors:** Adrien T. Stanley, Michael R. Post, Clay Lacefield, David Sulzer, Maria Concetta Miniaci

**Affiliations:** 1 Departments of Psychiatry, Neurology, and Pharmacology, Columbia University Medical Center, New York, NY USA; 2 Department of Pharmacy, University of Naples Federico II, Naples, Italy

**Keywords:** Synaptic plasticity, Learning and memory

## Abstract

The modulation of dopamine release from midbrain projections to the striatum has long been demonstrated in reward-based learning, but the synaptic basis of aversive learning is far less characterized. The cerebellum receives axonal projections from the locus coeruleus, and norepinephrine release is implicated in states of arousal and stress, but whether aversive learning relies on plastic changes in norepinephrine release in the cerebellum is unknown. Here we report that in mice, norepinephrine is released in the cerebellum following an unpredicted noxious event (a foot-shock) and that this norepinephrine release is potentiated powerfully with fear acquisition as animals learn that a previously neutral stimulus (tone) predicts the aversive event. Importantly, both chemogenetic and optogenetic inhibition of the locus coeruleus-cerebellum pathway block fear memory without impairing motor function. Thus, norepinephrine release in the cerebellum is modulated by experience and underlies aversive learning.

## Abstract (web summary)

The role of norepinephrine in cerebellum during fear learning is not fully understood. Here the authors investigate the role of a pathway from locus coeruleus to the cerebellum that contributes to fear memory formation.

## Introduction

Traumatic events forge learned associations of sensory signals with aversive outcomes and can lead to excessive conditioned fear responses as well as anxiety and posttraumatic stress disorders^1^ (PTSD). While a central role for dopamine release from ventral midbrain axons in the striatum has long been established for forms of reward-based learning^2,3^, the circuitry underlying aversive learning has been controversial^4^, with evidence endorsing roles for both a subset of ventral midbrain dopamine neurons^5,6^ and for locus coeruleus-norepinephrine (LC-NE) neurons, which possess axons that project widely to many brain regions^7–9^.

NE release from LC projections has been implicated in “fight-or-flight” responses to stressful stimuli and stimulus-associated fear. Aversive stimuli and stress-related behaviors trigger NE release from LC axons in the hypothalamus during forced swimming and tail suspension^10^ and within the amygdala following foot-shock, where it activates β-adrenergic receptors^11–13^. Indeed, systemic β-adrenergic receptor antagonists or α2-AR adrenergic receptor agonists impair fear acquisition^14–16^.

LC axons also project to the cerebellum (CB)^17,18^, a brain region implicated in emotional associative learning^19–21^ via its outputs to the amygdala, periaqueductal gray, hypothalamus and pre-frontal cortex^22–24^. Notably, stimulation of the CB vermis produces freezing and bradycardia, whereas lesions of the vermis (lobules IV and V) inhibit autonomic and behavioral fear responses^25–28^. However, roles for NE in the CB in fear learning have not been directly explored.

Here, we report that conditioned aversive learning relies on an experience-dependent increase of NE release in the CB when a conditioned stimulus (CS) becomes associated with an aversive unconditioned stimulus (US). Importantly, this learned potentiation of NE release is correlated with freezing, and the disassociation of the predictive US with LC axonal or cell body activity impairs acquisition of learned freezing, without affecting motor functions. The discovery that plastic changes in the synaptic activity of LC-NE projections to the cerebellum play a central mechanism in aversive learning may be key to deciphering how animals learn to avoid threats, as well as assist in identifying the causes of anxiety, posttraumatic stress, and impulse control disorders.

## Results

### Imaging locus coeruleus projections to cerebellum

Previous studies have demonstrated that TH-immunoreactive fibers are densely distributed to all cerebellar lobules, laminae and nuclei^29^, with the highest expression found in anterior vermis and cerebellar nuclei^21^.

**Fig. 1:**
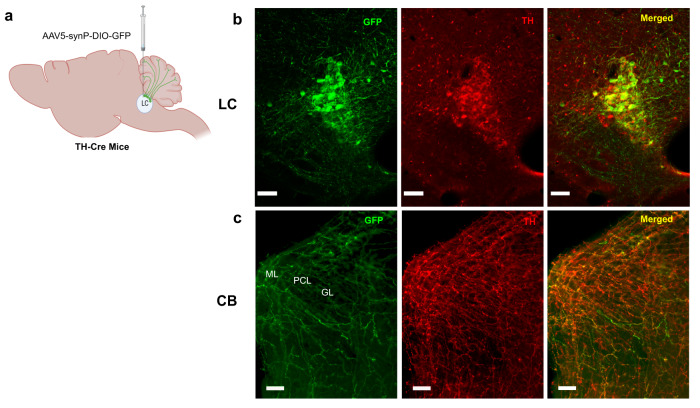
The cerebellum exhibits a widespread expression of NE fibers. **a** Schematic illustration of stereotaxic injection of AAV5-GFP into the LC of TH-Cre mice. Four to six weeks after AAV-delivery, immunostaining revealed expression of TH and GFP in the LC neurons (created with BioRender.com) (**b**) and their axonal projections to CB vermis (**c**); PCL Purkinje cell layer, GL granular layer, and ML molecular layer. One representative image of 6 independent experiments is shown in b and c. Scale bar, 100 μm.

To image LC projections within the cerebellum, we used a Cre expressing mouse line driven by the promoter for tyrosine hydroxylase (TH), the rate-limiting enzyme for catecholamine synthesis. In these mice, the injection of Cre-dependent AAV-GFP into the LC (Fig. 1a) resulted in GFP expression in LC cell bodies and axonal fibers that overlapped with the TH immunolabel (Fig. 1b). Consistent with prior reports of extensive LC projections to the cerebellum^21,29^, we observed diffuse GFP-labeled fibers in the cerebellar cortex that colocalized with TH^+^ axons (Fig. 1c).

**Fig. 2:**
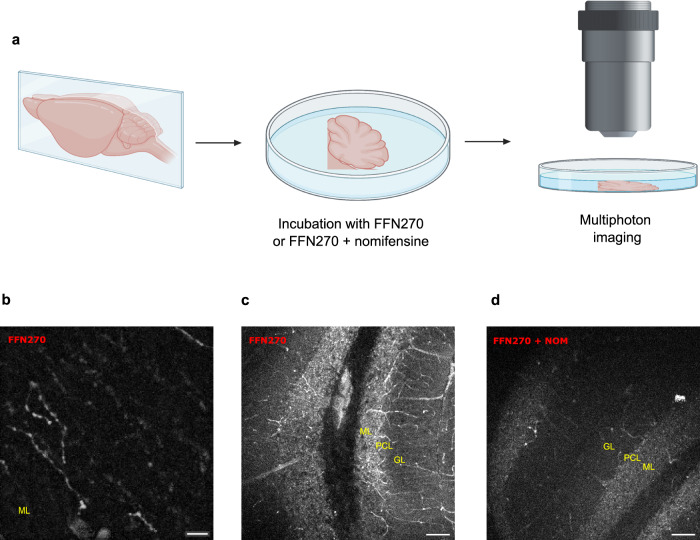
FFN270 labels NE axons in the cerebellum. **a** Schematic of cerebellar brain slice preparation and incubation with FFN270 with or without nomifensine (created with BioRender.com). **b**, **c** Representative 2-photon microscopy images of FFN270 (10 μM) loaded into the NE axons of acute cerebellar slices (lobule IV; one representative image of 4 independent experiments is shown in **b** and **c**). **d** The FFN270 axonal labeling was inhibited by nomifensine (lobule IV; NOM, 5 μM; representative of 3 independent experiments). PCL Purkinje cell layer, GL granular layer, and ML molecular layer. The scale bar is 5 μm in (**b**); 100 μm in (**c** and **d**).

To image norepinephrinergic synaptic vesicles in LC axons, we incubated acute cerebellar slices with the fluorescent false neurotransmitter FFN270^30^, an NE analog that is a substrate for the norepinephrine transporter (NET) that accumulates extracellular NE into the cytosol, and VMAT2, which mediates the uptake of monoamines including NE into synaptic vesicles (Fig. 2a). Using two-photon microscopy, we observed punctate FFN270 in *en passant* varicosities in the cerebellar cortex (Fig. 2b, c). Consistent with uptake by NET, FFN270 axon label of these structures was inhibited by the NET inhibitor nomifensine (5 μM; Fig. 2d). Thus, the presence of NE axons in the cerebellum was corroborated by TH immunolabel, TH-Cre-dependent GFP label from injection into the LC, and accumulation of a fluorescent NE derivative that is a substrate for NET and VMAT2.

### Fear recall elicits cerebellar release of NE detected by the GRAB_NE_ sensor

**Fig. 3:**
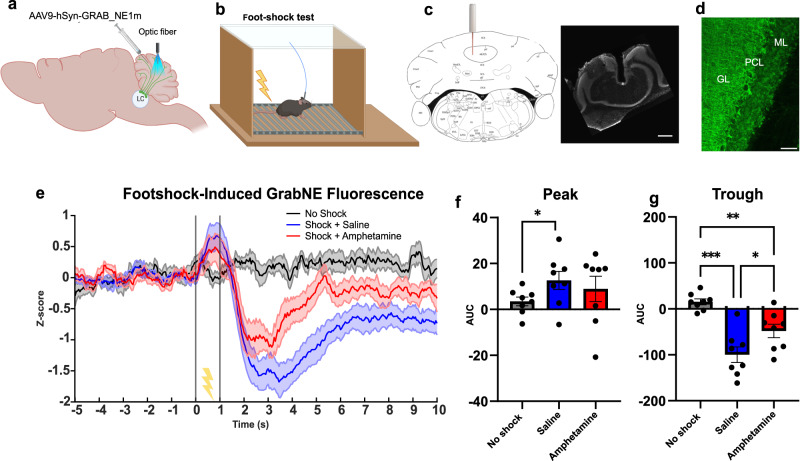
Effect of foot-shock on the NE release in the cerebellum. **a** AAV9-GRABNE was injected in the mouse cerebellar vermis and the optical fiber was implanted above the injection site (created with BioRender.com). **b** Mice were individually placed in the chamber and received 10 foot-shocks (0.5 mA, 1 s) with an inter-shock interval of 60 s (created with BioRender.com). **c** On the left, stereotaxic location of the optic fiber; based on Franklin and Paxinos’s mouse brain atlas (2007). On the right, histological section of the mouse cerebellum stained with DAPI showing the optic fiber placement (the image is representative of 8 independent experiments). Scale bar is 500 μm. **d** Representative image of 8 independent experiments of GRABNE expression in the cerebellum. PCL Purkinje cell layer, GL granular layer, and ML molecular layer. Scale bar is 100 μm. **e** Time course of GRABNE fluorescence, expressed as Z-score, in response to foot-shock under control condition (saline) and following systemic administration of amphetamine (10 mg/kg, i.p.), *n* = 8 mice. **f**, **g** Response to foot-shock measured as area under the curve (AUC) from 0 to 1.4 s (two-sided paired *t*-test no shock vs shock (saline) *p* = 0.0443; *n* = 8 mice) and from 1.4 to 5 s (two-sided paired t-test no shock vs shock (saline) *p* = 0.0001; *n* = 8 mice). Systemic administration of amphetamine (Amph) had no effect on the peak (AUC from 0 to 1.4 s: one-way ANOVA for treatment *F*(1.657, 11.6) = 1.653, *p* = 0.2327.) but induced a significant decrease in fluorescence relative to the trough (AUC from 1.4 to 5 s: one-way ANOVA for treatment *F*(1.962, 13.73) = 30.38, *p* < 0.0001, followed by Tukey test for multiple comparisons shock vs shock + amphetamine *p* = 0.0171); *n* = 8 mice per treatment. Data are presented as mean values ± SEM, corresponding to bars or shaded regions. Source data are provided as Source Data file.

To analyze NE release in the cerebellum, we used the fluorogenic NE reporter, GRAB_NE_^10^. We injected C57BL/6 mice with an AAV that express GRAB_NE_ in the CB and implanted an optic fiber above the injection site (Fig. 3a, c). After 6-8 weeks, mice were placed in a chamber and administered ten foot-shocks (0.5 mA intensity, 1 s duration) at 60 s intervals while recording the GRAB_NE_ fluorescence (Fig. 3b, d). A shown in Fig. 3e–g, the signal was biphasic with a peak at 0.8 s (±0.76) followed by a trough at 3.2 s (±0.29): the levels of NE at baseline cannot be determined using this technical approach.

The administration of the psychostimulant amphetamine (10 mg/kg), a competitive inhibitor of NET^30,31^, prior to foot-shock presentation attenuated the GRAB_NE_ signal trough by 52.9% (±12) and had no effect on the peak (Fig. 3e–g). We therefore conclude that foot-shock drives an acute short duration increase in NE release followed by a prolonged decrease in ambient NE which encompasses NET reuptake and diffusion of the neurotransmitter away from the recorded sites.

**Fig. 4:**
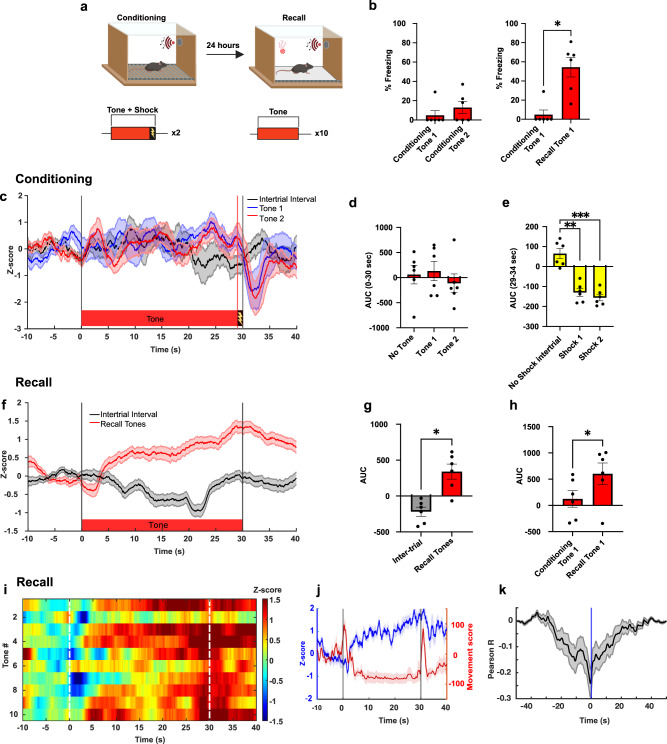
Threat conditioned stimulus triggers NE release in the cerebellum. **a** During fear conditioning, mice received two CS–US pairing trials. After 24 h, mice were exposed to 10 tones alone in a different context (recall) (created with BioRender.com). **b** Left graph: Percent time freezing during conditioning (two-sided paired *t*-test *p* = 0.1041; *n* = 6 mice). Right graph: Percent time freezing in response to the first conditioning tone and the first recall tone (two-sided paired *t*-test *p* = 0.0173; *n* = 6 mice). **c** Time course of average GRAB_NE_ fluorescence, expressed as Z-score, in response to 2 conditioning tone-shock presentations and intertrials (*n* = 6 mice). **d**, **e** Conditioning tones did not cause a significant change in GRAB_NE_ fluorescence (AUC 0-30 s: one-way ANOVA *F*_(1.442, 7.212)_ = 0.662 relative to intertrial; *n* = 6 mice) while foot-shock induced a significant fluorescence decrease (AUC 29-34 s: *F*_(1.574, 7.869)_ = 39.28, *p* = 0.0001 relative to intertrial; *n* = 6 mice). **f** Time course of GRAB_NE_ fluorescence across the 10 recall tones and the intertrials (*n* = 6 mice). **g** GRABNE fluorescence across 10 recall tones relative to the intertrials (two-sided paired *t*-test *p* = 0.0117; *n* = 6 mice). **h** GRAB_NE_ fluorescence change during the first CS presentation in the recall test vs. the first CS presented in the conditioning (two-sided paired *t*-test *p* = 0.0258; *n* = 6 mice). **i** Heat map showing the mean GRAB_NE_ fluorescence response to each of the 10 recall tones (*n* = 6 mice). **j** GRAB_NE_ fluorescence and movement score in response to recall tone in one representative mouse. Red trace represents movement score while blue trace represents fluorescence. The two movement peaks occur at the beginning and the end of the recall tones. **k** Pearson cross-correlation (*n* = 6 mice) between GRAB_NE_ fluorescence and movement score in response to recall tone. Data are presented as mean values ± SEM, corresponding to bars or shaded regions. Source data are provided as Source Data file.

We then examined whether the release of NE into the cerebellum is modulated by threat learning and memory induced by pairing CS tone and US foot-shock. On Day 1, mice were placed in a chamber and administered two 30 s tones, both of which co-terminated with a 1 s duration foot-shock (Fig. 4a). Twenty-four hours later, mice exposed to 10 tones with no foot-shocks in a novel context exhibited freezing, demonstrating cued threat memory formation (Fig. 4b; Supplementary Fig. 1a).

Analysis of the GRAB_NE_ fluorescence showed that during training, CS tones produced no change in NE signal (Fig. 4c, d), and consistent with the above results, both foot-shocks elicited a delayed 2.5-fold decrease in fluorescence at 3.1 s (±0.16) (Fig. 4c, e). On day 2, the presentation of a recall tone in the absence of foot-shock increased the NE signal relative to the intertrial (Fig. 4f, g, i; *t*-test *p* < 0.05; Supplementary Fig. 1b) which was 4.8-fold higher than the fluorescence measured during the tone presentation on Day 1 (Fig. 4h; *p* < 0.05). The GRAB_NE_ fluorescence activity during the Day 2 recall tone was negatively correlated with the overall movement (Fig. 4j, k). These results demonstrate that behavioral conditioning converted a neutral sensory cue that originally had no effect on NE release to one that elicits NE release in the cerebellum.

### Selective inhibition of LC-CB projections suppresses conditioned fear response

**Fig. 5:**
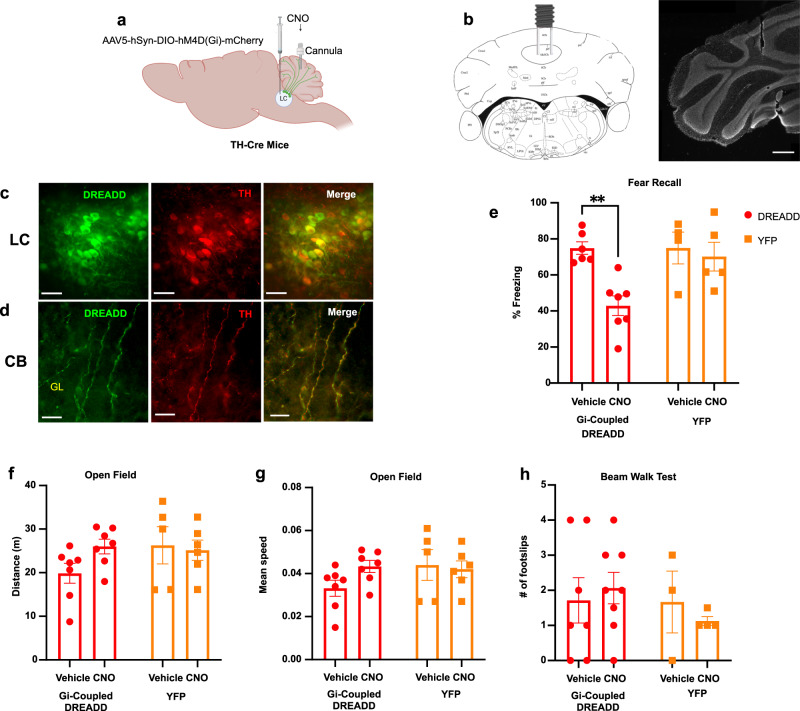
Inhibition of LC-NE projections to the CB impairs auditory fear conditioning. **a** Inhibitory DREADD hM4D(Gi)-mCherry or control YFP virus were bilaterally injected in the LC of TH-Cre mice. CNO (300 nM) or saline was delivered into the CB vermis (created with BioRender.com). **b** DAPI-stained cerebellar slice showing the cannula placement (right) and corresponding stereotaxic atlas page (left). Scale bar, 500 μm. The image is representative of at least four replicates per experimental conditions. **c**, **d** Gi-DREADD (green) and TH (red) double labeling of LC neurons and their projections to CB. GL granular layer. Scale bar, 50 μm. Each image is representative of at least four replicates per experimental conditions. **e** DREADD-mediated inhibition of LC-CB projections during fear conditioning reduced the freezing response to the recall tone (two-way ANOVA followed by Sidaks test: treatment factor (*F*(1, 18) = 8.376, *P* = 0.0097), viral expression factor (*F*(1, 18) = 4.632, *P* = 0.0452), interaction factor (*F*(1, 18) = 4.589, *P* = 0.0461); *n* = 7 mice treated with hM4Di/CNO, *n* = 6 mice treated with hM4Di/vehicle, *n* = 5 mice treated with YFP/CNO, *n* = 4 mice treated with YFP/vehicle. Measurement of the total distance (**f**) and speed (**g**) in the open field revealed no difference between hM4Di/CNO mice with respect to control mice (YFP and vehicle); *n* = 7 mice treated with hM4Di/CNO, *n* = 7 mice treated with hM4Di/vehicle, *n* = 6 mice treated with YFP/CNO, *n* = 5 mice treated with YFP/vehicle. **h** Number of footslip errors in narrow beam walking test showed no significant motor coordination impairment in hM4Di/CNO mice compared to control mice (YFP and vehicle); *n* = 8 mice treated with hM4Di/CNO, *n* = 7 mice treated with hM4Di/vehicle, *n* = 4 mice treated with YFP/CNO, *n* = 3 mice treated with YFP/vehicle. Data are presented as mean values ± SEM. Source data are provided as Source Data file.

We next investigated whether changes in cerebellar NE release in the cerebellum influences fear learning using chemogenetic and optogenetic approaches which were previously demonstrated to block the activity of noradrenergic axons^32,33^. To test this, Th-Cre mice received bilateral injections of AAV5-hSyn-DIO-hM4D(Gi)-mCherry virus into the LC to express the Gi-coupled inhibitory hM4Di receptor in TH^+^ neurons (Fig. 5a–d). Control mice were injected with YFP virus into the LC. Six-to-eight weeks later, the mice were administered local injections of the hM4Di agonist clozapine N-oxide (CNO) or vehicle via cannula in the cerebellum, 1 h prior to the fear conditioning on Day 1. On Day 2, when tones were administered without foot-shocks, hM4Di mice who received CNO displayed less freezing in response to the tone than control vehicle or YFP mice (Fig. 5e; Supplementary Fig. 2a). These results demonstrate that activity in LC-CB projections contributes to fear learning.

We next examined the effect of chemogenetic inhibition of LC projections to the cerebellum on general locomotor activity and motor coordination in the open field and beam walking test. As shown in Fig. 5f, g, there was no significant difference between groups in the total distance traveled and average speed for the four different groups of mice during the 10 min open field test. Similarly, the beam walking assay revealed no significant difference between groups of mice in the numbers of footslips while crossing the beam (Fig. 5h). These results demonstrate that silencing LC-CB projections does not alter general locomotor activity, indicating that the effect on fear learning was not due to locomotor changes.

**Fig. 6:**
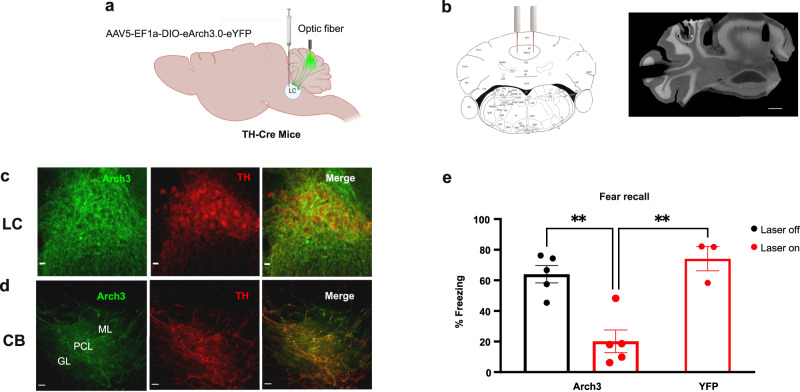
Inhibition of LC-NE projections to the CB impairs auditory fear conditioning. **a** Arch3-YFP was bilaterally injected into the LC of TH-Cre mice and optic fibers were bilaterally implanted in the CB vermis. **b** Stereotaxic and histology location of the optical fibers. On the left is a picture from the mouse brain atlas (Franklin and Paxinos; 2007). On the right is an image of a cerebellar slice showing the fiber placement in the cerebellum. Scale bar, 500 μm. The image is representative of at least four replicates per experimental conditions. **c**, **d** Arch3-YFP and TH expression in LC neurons and their axon terminals in the CB. PCL Purkinje cell layer, GL granular layer, and ML molecular layer. Scale bar, 100 μm. Each image is representative of at least four replicates per experimental conditions. **e** Optogenetic inhibition of LC axons in the CB during CS–US presentation suppressed the freezing response to recall tone (one-way ANOVA *F*(2, 10) = 16.87, *p* = 0,0006; Tukey’s post hoc test: Arch3-laser on versus YFP-laser on, *p* = 0.0013; Arch3-laser on versus Arch3-laser off, *p* = 0.002; Arch3-laser off versus YFP-laser on, *p* = 0.62; *n* = 5 mice treated with Arch3-laser on, *n* = 5 mice treated with Arch3-laser off, *n* = 3 mice treated with YFP-laser on). Data are presented as mean values ± SEM. Source data are provided as Source Data file.

To test whether the activity of cerebellar LC axons during the precise CS/US association step is required for fear learning, we injected a virus expressing the inhibitory channelrhodopsin eArch3 (AAV5-Ef1a-DIO-eArch3.0-EYFP) into the LC and bilaterally implanted fiber optics into the cerebellum (Fig. 6a–d). Mice injected with the YFP virus into the LC provided controls. On Day 1 of fear conditioning, a light stimulus was delivered through the fibers to inactivate the LC axons only during the time mice received the two tone-foot-shock pairings in the chamber. When the mice were tested on Day 2 for recall, we found that this brief optogenetic inhibition of LC-CB projections strongly decreased fear memory (Fig. 6e; Supplementary Fig. 2b) suggesting that LC neuronal activity within the cerebellum during tone-shock pairing is required for effective fear learning. In addition, we observed that the optogenetic inhibition of the NE axons in the cerebellum during the presentation of the recall tones on Day 2 induced a significant reduction of freezing behavior (see Supplementary Fig. 3). These results provide strong evidence that learned NE release in the cerebellum is required for the expression of freezing during recall of the fear memory. We acknowledge that the precise timing of LC activity and duration of enhanced NE signaling required for fear learning within the 30 s US-CS training paradigm is unknown and will require additional experimentation to address.

## Discussion

Here we identify a LC-CB pathway instrumental for fear memory. We find that the learned acquisition of an auditory CS association with a noxious event elicits the release of NE in the cerebellar vermis. The learned NE signal is correlated with freezing in response to the CS, indicating a synaptic basis for aversive learning. Importantly, the data indicate that the LC-CB pathway is critical for fear memory formation since its inhibition via chemogenetic and optogenetic approaches blocks fear recall. These results highlight a role for the LC in influencing the activity of target regions in the presence of a salient stimulus.

How does the cue-induced increase in NE release affect fear behavior? We have previously shown that NE promotes long-term potentiation (LTP) at the parallel fiber (PF)-PC synapse by lowering the threshold of LTP induction via β-ARs activation^34^. PF-PC LTP was suggested as a synaptic mechanism required for the formation of fear memories, since it occurs in slices of cerebellar vermis (lobules V and VI) obtained from fear conditioned animals but not from animals that are naive or receive unpaired presentations of the tone and shock^35^. The increased PF-PC synaptic efficacy is likely mediated by a postsynaptic mechanism that requires an activation of cAMP-PKA signaling^34^ that enhances the phosphorylation of AMPA receptors to promote their insertion into the synaptic membrane^36^. The learning-induced potentiation of NE response to CS that we report may serve to refine the PC output to deep cerebellar nuclei to control motor and cognitive behavior. Indeed, previous reports indicate that the CB vermis regulates freezing behavior via the fastigial (medial) cerebellar nucleus (FN) which projects to the ventrolateral periaqueductal gray^24^ (vlPAG). The vlPAG in turn projects to the magnocellular reticular nucleus, which excites spinal cord motor neurons responsible of the freezing in response to threating stimuli^25^. The vermis-vIPAG-magnocellular circuit is implicated in fear learning since both optogenetic and chemogenetic manipulations of the FN-vlPAG pathway during different phases of a fear conditioning paradigm affect fear memory formation^24,37^. We do not exclude that the lateral cerebellar hemispheres contribute to the NE modulation of fear memory, and recent reports indicate that LC provides a large projection to the lateral cerebellar nuclei that are involved in associative learning and cognitive functions^21,38^.

As reported independently^21^, we found that the inhibition of the LC-CB projections does not alter motor behavior and motor learning. It is possible that that the local administration of CNO may not affect NE levels in distal cerebellar regions that regulate locomotor and balance beam behaviors. An alternative possibility is that the fear conditioned stimulus may elicit a strong phasic response from LC neurons resulting in a large release of NE in cerebellum that may be significantly affected by the local chemogenetic/optogenetic inhibition, unlike the tonic release of NE that occurs during locomotor and balance beam testing.

Several studies indicate that LC neurons send and receive projections from brain regions such as the amygdala and hippocampus that are implicated in specific aspects of fear learning. The activation of LC-NE inputs to the BLA may be important during cued fear conditioning, since the optogenetic activation of this pathway promotes conditioned aversion and anxiety-like behavior through β-AR activation^12^. Photostimulation of LC projections in the dorsal hippocampus during contextual fear conditioning increases fear recall, suggesting that the LC innervation of the hippocampus is involved in the acquisition and consolidation of contextual fear^39^. As cerebellar lesions are reported to exert greater effects on cued fear memory than contextual fear memory^27,40,41^, and that recall of strong cued fear memories is prevented by the inactivation of amygdala and cerebellum, it is likely that cerebellum plays an essential role in cued fear memory processing. How LC neurons recruit and coordinate the neural circuits responsible for emotional associative learning remains an open question.

Our finding has important clinical implications for individuals experiencing trauma- and stress-related disorders, such as PTSD, characterized by an intense fear response^42^. Functional neuroimaging studies have revealed an increased activation of the cerebellar vermis in PTSD patients re-experiencing the traumatic event^43^, while a decreased activation of the cerebellum was associated with PTSD symptom improvement^44^. Moreover, PTSD patients exhibit an increased resting-state functional connectivity of the anterior cerebellar vermis with the amygdala and the periaqueductal gray compared to healthy control^45,46^.

Given the involvement of NE hyperactivity in PTSD^47^ and the significant influence of the NE system on cerebellum and fear memory formation, inhibiting the action of NE via β-adrenergic receptor antagonists might minimize the impact of cerebellar activity on fear response. This notion is supported by evidence that administration of the β-antagonist propranolol a few hours after trauma exposure^48^ or while re-experiencing the trauma memories alleviates PTSD symptoms^49^.

Aversive learning is also an important component of impulse control^50^, as it provides recognition of a harmful situations and triggers appropriate motor responses intended to avoid them. A loss of NE releasing LC neurons occurs early in several neurodegenerative disorders, including Alzheimer and Parkinson diseases^51^ and has been suspected to contribute to the impairment of cognitive functions including memory, attention, and arousal observed in these patients^52,53^. Parkinson’s patients under dopamine replacement therapy including L-DOPA may develop impulse control disorder, characterized by the inability to control impulsive destructive or maladaptive behaviors^54,55^. The reinstatement of reward-associated learning nigrostriatal circuitry by L-DOPA or dopamine receptor agonists without a corresponding therapy for aversion-associated learning in LC-cerebellar circuits may underlie the basis for this behavioral disorder.

## Methods

### Experimental models and subject details

All experimental procedures were approved by the Columbia University Institutional Animal Care and Use Committee (IACUC, AABI2605). All mice in this study were on a C57BL/6J background, typically group housed with littermates of the same sex, and provided access to food and water ad libitum. Mice were kept on a reverse light/dark cycle (lights off at 7:00, lights on at 15:00) and the behavioral tests were conducted during the dark phase. Experimental groups contained both male and female mice >8 weeks of age. Since the conditioned fear response was not significantly different between untreated males and females (see the Source data file), the sex was not considered in the study design and analysis. TH-IRES-Cre^+/−^ mouse line was obtained from Jackson Laboratory (Bar Harbor, ME, USA) and maintained by backcrossing to C57/B6J mice. The study included six Th-Cre mice (four males and two females) injected with AAV5-synP-DIO-eGFP-WPRE-hGH virus, seven C57BL/6J mice (four females and three males) for the FFN270 experiment, twenty-four mice C57BL/6J mice (eighteen females and six males) injected with AAV9-GRABNE, fifteen Th-Cre mice injected with DREADD hM4D(Gi)-mCherry (seven females and eight males) vs 11 Th-Cre mice (three females and eight males) injected with the control YFP virus for the chemogenetic inhibition of LC axons in the CB during conditioning, ten Th-Cre mice (six females and four males) injected with Arch3-YFP virus vs 3 Th-Cre mice (two females and one male) injected with the control YFP virus for the optogenetic inhibition of LC axons in the CB during conditioning, seven Th-Cre mice injected with Arch3-YFP virus for the optogenetic inhibition of LC axons in the CB during recall. Mice were 8-10 weeks old at the start of the study.

### Viruses

For immunostaining of LC fibers in the CB, 230 nl of AAV5-synP-DIO-eGFP-WPRE-hGH (Addgene,1*10^13 vg/ml) was injected into the LC of TH-Cre mice. For fiber photometry experiments targeting the CB vermis, 230 nl of AAV9-hSyn-GRAB_NE1m (Addgene,1 × 10^13^ vg/ml) was unilaterally injected in C57BL/6J mice. For optogenetic and chemogenetic experiments, 230 nl of AAV5-EF1a-DIO-eArch3.0-eYFP (Addgene,1 × 10^13^ vg/ml) and AAV5-hSyn-DIO-hM4D(Gi)-mCherry (Addgene,1 × 10^13^ vg/ml) were injected into the LC of TH-Cre mice, respectively; AAV5-EF1a-DIO-eYFP (230 nl, Addgene,1 × 10^13^ vg/ml) was injected into the LC of TH-cre mice used as controls.

### Surgical procedures

Injection and implant surgeries were performed under 2% isoflurane anesthesia using a Kopf stereotaxic apparatus. A small incision was made in the scalp and burr holes were drilled in the skull at the appropriate stereotaxic coordinates, relative to bregma: AP: −6.7, ML:0.75, DV:1 for CB anterior vermis; 5.45 AP, 1.3 ML, 3.8 DV for LC. Viruses were infused at a rate of 23 nl/min using a glass micropipette, backfilled with mineral oil, and connected to a Nanoject II (Drummond Scientific Co., Broomall, PA, USA). The injection pipette was slowly withdrawn 5 minutes after the end of infusion. For fiber photometry experiments, GRAB_NE_ reporter virus was injected into the CB vermis and optic fiber (Becker & Hickl) was implanted 0.1 mm above the target injection site. For optogenetic experiments, the AAV5-EF1a-DIO-eArch3.0-EYFP or eYFP virus was injected into the LC and optic fibers were bilaterally implanted in the CB vermis in order to provide wider coverage of the CB. For chemogenetic experiments, M4-Gi-DREADD virus or eYFP was injected into the LC and 26-gauge guide cannulas (Roanoke, VA, USA) cannulas were bilaterally implanted in the CB vermis and drugs were infused via a 33-guage internal cannula that extended 0.5 mm beyond the cannula tip (Roanoke, VA, USA). The bilateral implants for optogenetics and chemogenetics were positioned 0.5 mm to the right and left of midline, while unilateral implants for optical fibers measuring GRAB_NE_ fluorescence were positioned 0.5 mm to the right of midline. All experiments were conducted at least 4 weeks after the surgery. The location of the implanted fibers and cannulas were verified with postmortem histology.

### Histology procedures and imaging

Mice were deeply anesthetized and transcardially perfused with 0.9% NaCl followed by 4% paraformaldehyde (PFA) in 0.1 M phosphate buffer (PB). Brains were removed and post-fixed overnight in 4% PFA in 0.1 M PB. Brains were then washed three times in 1× phosphate buffered saline, cut into 40 μm sections using a VT1200 vibratome (Leica Biosystems) and stored in cryoprotectant (0.1 M PB, 30% glycerol, 30% ethylene glycol) at −20 °C. For immunofluorescence analysis, sections were washed in TBS three times and then blocked and permeabilized for 1 h at room temperature with 10% normal donkey serum (Jackson ImmunoResearch) and 0.1% Triton-X in TBS. Sections were then incubated overnight at 4 °C with primary antibodies, chicken anti-green fluorescent protein (1:500, Abcam) and rabbit anti-tyrosine hydroxylase (1:500, Millipore Sigma), in 2% normal donkey serum and 0.1% Triton-X in TBS. Sections were then washed in TBS three times and incubated for 2 hours in secondary antibodies, donkey/goat anti-chicken 488 (1:500) and donkey/goat anti-rabbit 647 (1:500), purchased from Invitrogen. Sections were washed again in TBS and mounted on microscope slides. Images were obtained using a fluorescence microscope (Olympus IX81 Microscope, Boston Industries, Inc. Walpole, MA, USA) equipped with 10×, 20×, and 40× objective lenses.

### Fiber photometry recording of GRAB_NE_ fluorescence during behavioral testing

Four weeks after surgery, GRAB_NE_ fluorescence was measured using time correlated single photon counting (TCSPC), custom built from Becker Hickl parts^56^ in mice while receiving a series of foot-shocks or while fear conditioning and recall testing took place. To measure fluorescent activity from GRAB_NE_, optic fiber implants (Becker & Hickl) were tethered to the TCSPC machine via a fiber optic patch cord. This patch cord delivered light from 473 nm picosecond pulsed laser to excite the GRAB_NE_ reporter and emitted photons were collected via the same patch cord. Photon counts were recorded by TCSPC Image software from Becker Hickl.

### Photometry analysis

GRAB_NE_ fluorescence was analyzed using custom scripts written in MATLAB. Photon counts were first converted into dF/F. To determine dF/F, we first calculated the background fluorescence at time *t*, B(*t*), by centering a sliding window (width 20 s) and determining the mean of the photon count within that window. The dF/F at time *t* is then calculated as [photon count at time *t* – *B*(*t*)/B(*t*)]. To quantify the fluorescence change (dF/F) in response to tone presentation, we considered a time frame of 50 s, corresponding to 10 s before tone onset, 30 s tone, and 10 s after tone offset. These fluorescence traces were then converted to z-score by subtracting the mean dF/F calculated across the 50 s trace and dividing by the standard deviation [(dF/F – mean dF/F)/(SD dF/F)]. To normalize traces to fluorescent activity that preceded tone onset, the average z-score of the 10 s preceding tone onset was subtracted from each trace. Tone traces for each mouse were then averaged together. As a control comparison, the same analysis was applied to a 50 s window in the intertrial interval. Area under the curve during tone presentation was calculated using the MATLAB “TRAPZ” function.

### Behavioral assays

For fear conditioning, mice were placed individually in a conditioning chamber which consisted of a clear plexiglass box (19 × 21 × 12.5 cm) enclosed in a wooden chamber to reduce external noise and visual stimulation. The box was equipped with a stainless-steel rod floor connected to a shock generator and a speaker connected to a tone generator. A video camera in front of the chamber enabled video recording. On day 1, each mouse was placed in a conditioning chamber and allowed to explore the testing chamber for 2 min. Following habituation, mice received two CS–US pairing trials with a 2 min intertrial interval^15^; each pairing consisted of a 30 s CS tone (80 dB, 3.5 KHz) co-terminating with a 1 s US foot-shock (0.5 mA). The mouse was then allowed to remain in the conditioning chamber for 2 min and then was returned to its original cage. Twenty-four hours later, mice were placed back into a modified chamber and tested by presenting 10 CSs with an intertrial interval ranging from 90 to 120 s. This chamber was the same box used for the conditioning session, but with peppermint odor and a smooth white plastic floor covering the rod flooring. The percentage of total time in which an animal exhibited freezing behavior was recorded and analyzed using a video tracking software (ANY-MAZE, Stoelting Co., Wood Dale, IL, USA). Freezing was defined as a defensive posture in which no movement except for respiration was observed under a threshold of 2 s of inactivity.

General locomotor activity was examined using the open field test^57^. Each mouse was placed in the center of the apparatus, consisting of a square area (40 × 40 cm) surrounded by white acrylic walls, and were allowed to explore freely for 30 minutes. The open field was divided into the inner zone (20 × 20 cm) and the outer zone. The total distance traveled and the time spent in the inner zone were recorded and analyzed using the ANY-MAZE software.

Motor coordination and balance were tested using the balance beam test. Mice were placed at the end of a 100 cm-long wood beam (1.0 cm wide), suspended above an open cage. Each animal was first pre-trained to walk across the beam twice. Mice were then trained to traverse the beam 5 times per day for 3 consecutive days. The number of times a hind paw slipped off the beam was measured, and the data were averaged.

### FFN and brain slice imaging

Experiments were performed on cerebellar slices of C57BL/6 mice at 8–9 weeks^30^. Each mouse was anesthetized with isoflurane, USP (Abbott Laboratories, Illinois, USA) and decapitated. The cerebellar vermis was removed and rapidly immersed in an ice-cold solution containing (in mM): 125 NaCl, 2.5 KCl, 2 CaCl_2_, 1 MgCl_2_, 1.25 NaH_2_PO_4_, 26 NaHCO_3_, 20 glucose, and the pH was maintained at pH 7.4 by bubbling with 95% O_2_—5% CO_2_. Parasagittal cerebellar slices (250 μm thickness) were cut using a Leica VT1200 vibratome (Leica Microsystems) at 4 °C. Slices were kept at room temperature in oxygenated (95% O_2_, 5% CO_2_) artificial cerebrospinal fluid (ACSF containing (in mM): 125 NaCl, 2.5 KCl, 25 NaHCO_3_, 1.25 NaH_2_PO_4_, 2 CaCl_2_, 1 MgCl_2_, 10 glucose, pH 7.3–7.4, 295–305 mOsm), and used within 1–5 h.

Each cerebellar slice was incubated in ACSF with the FFN270 (10 μM) for 30 min and then washed for 30 min before imaging. For the NET inhibition experiments, slices were incubated with 5 μM nomifensine for 30 min and then in 10 μM FFN270 with 5 μM nomifensine still present, and finally washed for 30 min with ACSF. Each slice was transferred to a QE-1 imaging chamber (Warner Instruments, Hamden, CT, USA), held in place by platinum wire and nylon, and superfused (1 ml/min) with oxygenated ACSF. The images were acquired using a Prairie Ultima Multiphoton Microscopy System (Prairie Technologies, Middleton, WI, USA) with a titanium-sapphire Chameleon Ultra II laser (Coherent) equipped with a 60× (0.9 NA) and 10× (0.3 NA) water immersion objectives. FFN270 was excited at 760 nm and 460(±) 25 nm light was collected. The 10× and 60× images were taken on a 1024 × 1024 pixel resolution and a dwell time of 8 μs/pixel. Relative steady state power was 6–8 mW.

### Chemogenetics

Chemogenetic experiments were performed as described^58^. CNO (0.3 µl, 300 nM in saline) or saline was delivered bilaterally to CB vermis (see coordinates above) via cannula-based microinfusions using a Hamilton syringe and a syringe infusion pump (Harvard Apparatus Model 11, Holliston, MA, USA) at a rate of 0.1 µl/min. At the end of each infusion, the internal cannula was left in place for at least 10 min to ensure diffusion, and mice were tested 30 min after removing the cannula. Each mouse received CNO before fear conditioning (on day 1) or before the motor tests. At that concentration and volume, the CNO stayed restricted in the cerebellar area^59^.

### Optogenetic

Optogenetic experiments were performed as described^60^. A green laser (532 nm, Laserglow) was used to stimulate Arch3 and induce optogenetic inhibition. The laser was connected to the implanted ferrule with an optic fiber cable (200 μm in diameter, 0.39 NA, Thor Labs) before the mouse was placed in the conditioning chamber. The laser power was set at 8–10 mW at the fiber tip. The laser was controlled by a Master 9 unit (A.M.P.I., Israel). To examine the effect of optogenetic inhibition of LC-NE projections during fear conditioning, the green light was delivered only during the tone-shock pairing trials of fear conditioning (on day 1) and was turned off during the intertrial interval. To examine the effect of optogenetic inhibition during fear recall, the green light was delivered only during the recall tones (on day 2) and was turned off during the intertrial interval.

### Statistical analysis

Statistical analyses were performed using GraphPad Prism 8 (GraphPad Software, San Diego, CA, USA). Data are presented as mean ± SEM. Paired and unpaired two-tailed Student’s t-tests were used to compare 2-group data, as appropriate. For comparison of two different conditions a two-tailed t-test was used; for multiple comparison one-way ANOVA was used followed by Tukey’s or Sidaks post hoc test, when appropriate. Data analyses were performed blind to the conditions of the experiments.

### Reporting summary

Further information on research design is available in the Nature Portfolio Reporting Summary linked to this article.

### Supplementary information

Supplementary Information

Reporting Summary

### Source data

Source Data

## Data Availability

The fiber photometry data generated in this study have been deposited in the Figshare database [10.6084/m9.figshare.23589747.v2]. All the data used in this study are included within the manuscript’s figures or provided in the supplementary information section and Source Data files. Source data, disaggregated by sex, are provided with this paper. Source data are provided with this paper.

## Code availability

The custom MATLAB scripts used for fiber photometry analysis is posted on github (https://github.com/DSulzerLab/ TCSPC_photometry_analysis) and raw data required for these scripts are posted on figshare (10.6084/m9.figshare.23589747.v2).
