# Supplementary material for: Norepinephrine release in the cerebellum contributes to aversive learning
Source: Nat Commun. 2023 Aug 10;14:4852. doi: 10.1038/s41467-023-40548-8 (PMC10415399; doi:10.1038/s41467-023-40548-8)
Supplement: Supplementary file 1 — Supplementary Information [file 41467_2023_40548_MOESM1_ESM.pdf]

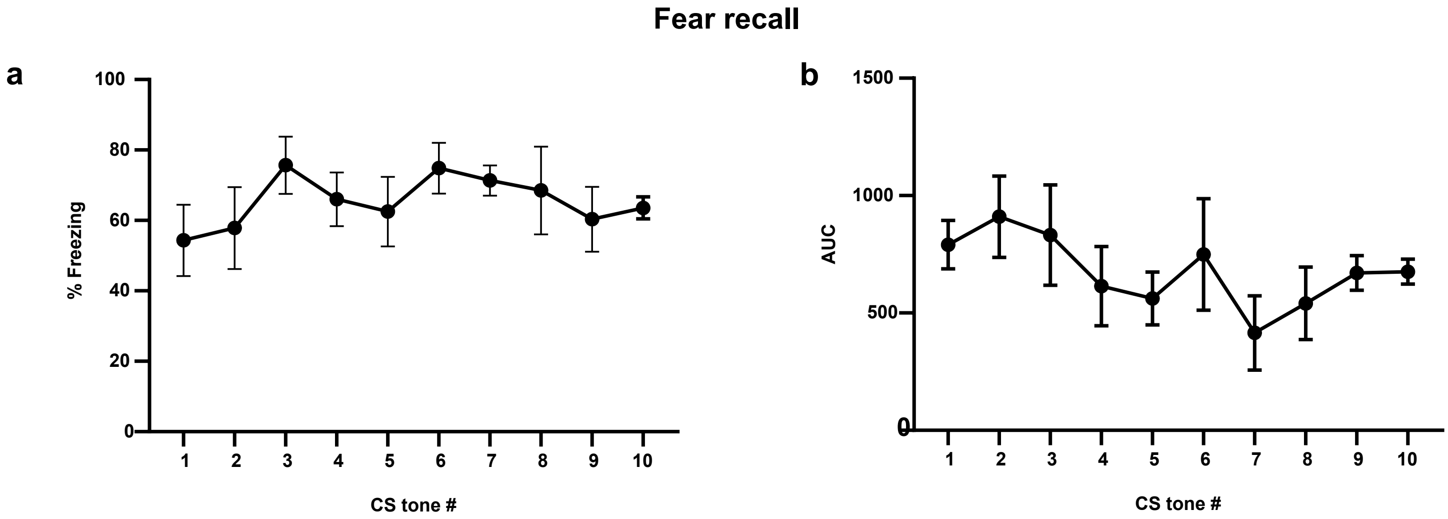

Suppl Fig. 1. Freezing and GRABNE fluorescence throughout the recall. a Percentage time freezing in response to the 10 recall tones (one-way ANOVA $F(9, 54) = 0.69$, $p = 0.71$; $n = 6$ mice). **b** Change of GRABNE fluorescence, expressed as AUC across the 10 recall tones (one-way ANOVA $F(9, 54) = 0.93$, $p = 0.50$; $n = 6$ mice). Data are presented as mean +/- SEM.

Fear conditioning

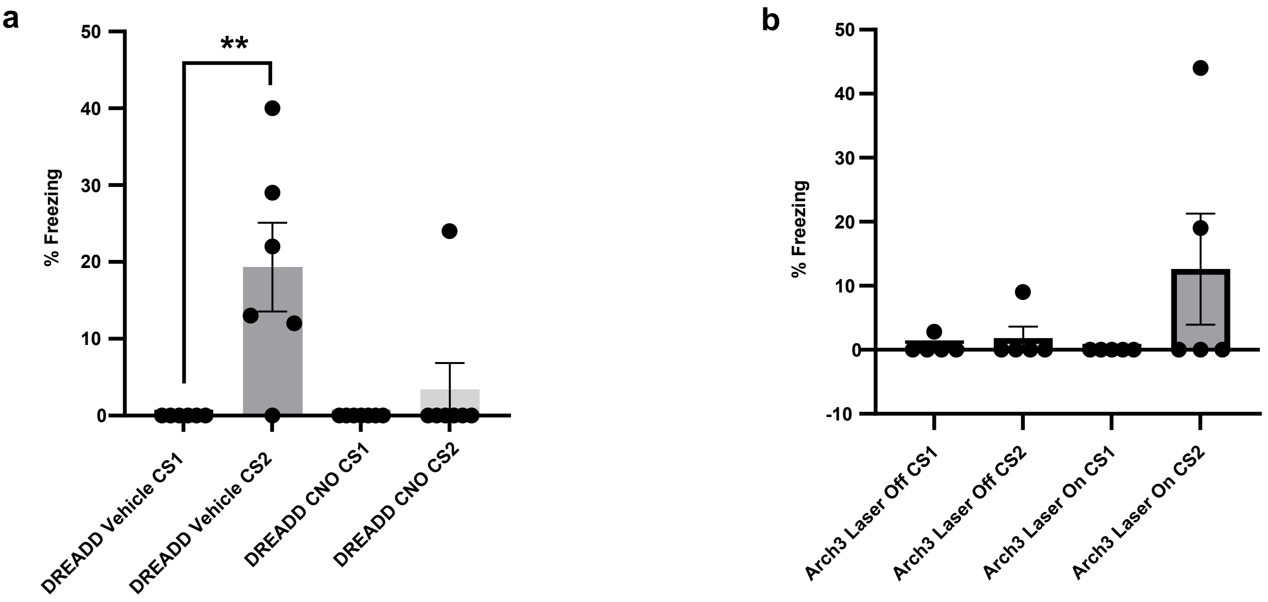

Suppl Fig. 2. Freezing response during fear conditioning. **a** Percentage time freezing in response to the two conditioning tones in DREADD hM4Di/CNO mice compared to DREADD hM4Di/vehicle mice (two-way ANOVA followed by Sidaks test: treatment factor (F(1, 22) = 12,37, P=0.0019), viral expression factor (F(1, 22) = 6.037, P=0.0224), interaction factor (F(1, 22) = 6,037, P=0.0224); DREADD vehicle CS1 vs CS2, p = 0.001; DREADD CNO CS1 vs CS2, p = 0.69; n = 7 mice treated with hM4Di/CNO, n = 6 mice treated with hM4Di/vehicle. **b** Percentage time freezing in response to the two conditioning tones in Arch3-laser on mice versus Arch3-laser off mice (two way Anova followed by Sidaks test: laser factor (F(1, 16) = 1.332, P=0.2654), treatment factor (F(1, 16) = 2.433, P=0.1383), interaction factor (F(1, 16) = 1.639, P=0.2186); n=5 mice treated with Arch3-laser on, n = 5 mice treated with Arch3-laser off. Data are presented as mean +/- SEM.

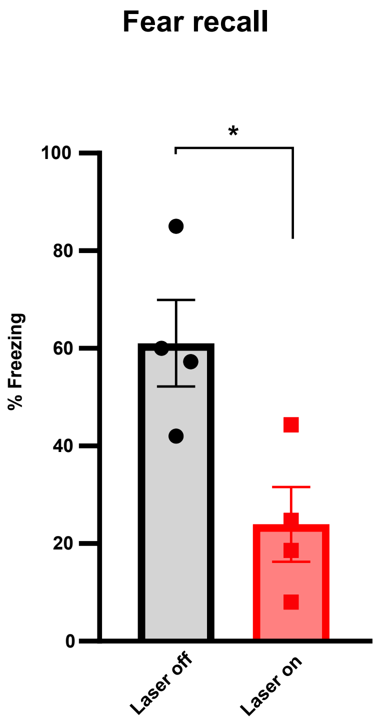

Suppl Fig. 3. Optogenetic inhibition of LC axons in the CB during recall reduces the freezing response to tones (two sided t-test, * p = 0.039; n = 4 mice). Data are presented as mean +/-SEM.
