# Supplementary material for: Norepinephrine release in the cerebellum contributes to aversive learning
Source: Nat Commun. 2023 Aug 10;14:4852. doi: 10.1038/s41467-023-40548-8 (PMC10415399; doi:10.1038/s41467-023-40548-8)
Supplement: Supplementary file 2 — Reporting Summary [file 41467_2023_40548_MOESM2_ESM.pdf]

nature portfolio

Corresponding author(s): Adrien T Stanley, Michael R Post, Clay O. Lacefield, David Sulzer, & Maria Concetta Miniaci

Last updated by author(s): Jul 3, 2023

Reporting Summary

Nature Portfolio wishes to improve the reproducibility of the work that we publish. This form provides structure for consistency and transparency in reporting. For further information on Nature Portfolio policies, see our Editorial Policies and the Editorial Policy Checklist.

Statistics

For all statistical analyses, confirm that the following items are present in the figure legend, table legend, main text, or Methods section.

n/a	Confirmed	
☐	☒	The exact sample size (*n*) for each experimental group/condition, given as a discrete number and unit of measurement
☒	☐	A statement on whether measurements were taken from distinct samples or whether the same sample was measured repeatedly
☐	☒	The statistical test(s) used AND whether they are one- or two-sided<br>*Only common tests should be described solely by name; describe more complex techniques in the Methods section.*
☒	☐	A description of all covariates tested
☒	☐	A description of any assumptions or corrections, such as tests of normality and adjustment for multiple comparisons
☐	☒	A full description of the statistical parameters including central tendency (e.g. means) or other basic estimates (e.g. regression coefficient) AND variation (e.g. standard deviation) or associated estimates of uncertainty (e.g. confidence intervals)
☐	☒	For null hypothesis testing, the test statistic (e.g. *F*, *t*, *r*) with confidence intervals, effect sizes, degrees of freedom and *P* value noted<br>*Give P values as exact values whenever suitable.*
☒	☐	For Bayesian analysis, information on the choice of priors and Markov chain Monte Carlo settings
☒	☐	For hierarchical and complex designs, identification of the appropriate level for tests and full reporting of outcomes
☐	☒	Estimates of effect sizes (e.g. Cohen's *d*, Pearson's *r*), indicating how they were calculated

Our web collection on statistics for biologists contains articles on many of the points above.

Software and code

Policy information about availability of computer code

Data collection	All behavioral data were collected using Any-maze Video Tracking System (version 4.99z). Real time fiber photometry signals were collected using a Time Correlated Single Photon Counting (TCSPC) system, custom built from Becker Hickl parts using Becker Hickl Single Photon Counting Software (version 9.75) (G. Cui et al., Nat. Protoc. 2014). Images were obtained using a fluorescence microscope (Olympus IX81 Microscope, Boston Industries, Inc. Walpole, MA, USA) or a Prairie Ultima Multi photon Microscopy System (Prairie Technologies, Middleton, WI, USA), as indicated.
Data analysis	Matlab (2021b) was used for fiber photometry analysis. Statistical analysis was performed using preset algorithms in Graph pad Prism (version 8). Figures were prepared in Inkscape (version 1.1). The custom MATLAB scripts used for fiber photometry analysis is posted on github [https://github.com/DSulzerLab/TCSPC_photometry_analysis] and raw data required for these scripts are posted on figshare [https://doi.org/10.6084/m9.figshare.23589747.v2].

For manuscripts utilizing custom algorithms or software that are central to the research but not yet described in published literature, software must be made available to editors and reviewers. We strongly encourage code deposition in a community repository (e.g. GitHub). See the Nature Portfolio guidelines for submitting code & software for further information.

Data

Policy information about availability of data

All manuscripts must include a data availability statement. This statement should provide the following information, where applicable:

- Accession codes, unique identifiers, or web links for publicly available datasets
- A description of any restrictions on data availability
- For clinical datasets or third party data, please ensure that the statement adheres to our policy

The fiber photometry data generated in this study have been deposited in the Figshare database [https://doi.org/10.6084/m9.figshare.23589747.v2]. All the data used in this study are included within the manuscript's figures or provided in the supplementary information section and Source Data files. Source data, disaggregated by sex, are provided with this paper.

Research involving human participants, their data, or biological material

Policy information about studies with human participants or human data. See also policy information about sex, gender (identity/presentation), and sexual orientation and race, ethnicity and racism.

Reporting on sex and gender	No human data was used in this study.
Reporting on race, ethnicity, or other socially relevant groupings	No human data was used in this study.
Population characteristics	No human data was used in this study.
Recruitment	No human data was used in this study.
Ethics oversight	No human data was used in this study.

Note that full information on the approval of the study protocol must also be provided in the manuscript.

Field-specific reporting

Please select the one below that is the best fit for your research. If you are not sure, read the appropriate sections before making your selection.

☒ Life sciences ☐ Behavioural & social sciences ☐ Ecological, evolutionary & environmental sciences

For a reference copy of the document with all sections, see nature.com/documents/nr-reporting-summary-flat.pdf

Life sciences study design

All studies must disclose on these points even when the disclosure is negative.

Sample size	No statistical methods were used to predetermine sample sizes for our experiments, but our sample sizes were based on prior literature demonstrating significance. Sample sizes for GrabNE fiber photometry experiments well exceeded those reported in previous publications in mouse (Feng et al. 2019). Sample sizes for DREADD and optogenetic experiments were comparable to prior studies (Carlson et al., 2021; Frontera et al., 2020; Morrissette et al., 2019; Huang et al., 2016; El-Gaby at al., 2016; Hormigo et al., 2016)
Data exclusions	No data were excluded
Replication	Chemogenetic and optogenetic experiments were performed with at least 2 separate cohorts of mice. Our main result showing reduced fear learning after inhibition of LC terminals in the CB was replicated in both chemogenetic and optogenetic approaches.
Randomization	For optogenetic and chemogenetic experiments, all mice were randomly assigned to control or treatment groups
Blinding	Experimenters collecting or analyzing the data blind to experimental conditions. Behavioral scores were assessed in Any-maze to avoid potential experimenter bias caused by human scoring.

Reporting for specific materials, systems and methods

We require information from authors about some types of materials, experimental systems and methods used in many studies. Here, indicate whether each material, system or method listed is relevant to your study. If you are not sure if a list item applies to your research, read the appropriate section before selecting a response.

Materials & experimental systems

n/a	Involved in the study
[ ]	[x] Antibodies
[x]	[ ] Eukaryotic cell lines
[x]	[ ] Palaeontology and archaeology
[ ]	[x] Animals and other organisms
[x]	[ ] Clinical data
[x]	[ ] Dual use research of concern
[x]	[ ] Plants

Methods

n/a	Involved in the study
[x]	[ ] ChIP-seq
[x]	[ ] Flow cytometry
[x]	[ ] MRI-based neuroimaging

Antibodies

Antibodies used

chicken anti-green fluorescent protein (1:500, Abcam, ab13970)
rabbit anti-tyrosine hydroxylase (1:500, Millipore Sigma, AB152)
goat secondary anti-chicken 488 (1:500, Invitrogen, A32733)
donkey secondary anti-rabbit 647 (1:500, Invitrogen, A-21206)

Validation

All antibodies were validated on the manufacturers web page:
chicken anti-green fluorescent protein (Abcam, ab13970): https://www.abcam.com/gfp-antibody-ab13970.pdf
rabbit anti-tyrosine hydroxylase (Millipore Sigma, AB152): https://www.emdmillipore.com/US/en/product/Anti-Tyrosine-Hydroxylase-Antibody,MM_NF-AB152#anchor_COA
goat secondary anti-chicken 488 (Invitrogen, A32733): https://www.thermofisher.com/order/genome-database/dataSheetPdf?producttype=antibody&productsubtype=antibody_secondary&productId=A-11039&version=223
donkey secondary anti-rabbit 647 (Invitrogen, A-21206): https://www.thermofisher.com/order/genome-database/dataSheetPdf?producttype=antibody&productsubtype=antibody_secondary&productId=A-21206&version=223

Animals and other research organisms

Policy information about studies involving animals; ARRIVE guidelines recommended for reporting animal research, and Sex and Gender in Research

Laboratory animals

All mice in this study were on a C57BL/6J background, typically group housed with littermates of the same sex, and provided access to food and water ad libitum. Mice were kept on a reverse light/dark cycle (lights off at 7:00, lights on at 15:00) and the behavioural tests were conducted during the dark phase. Adult male and female mice > 8 weeks of age were used for all experiments. Experimental groups contained equal numbers of male and female mice. TH-IRES-Cre+/− mouse line was obtained from Jackson Laboratory (Bar Harbor, ME, USA) and maintained by backcrossing to C57/B6J mice.

Wild animals

No wild animals were used in this study.

Reporting on sex

Source data, disaggregated by sex, are provided with this manuscript. Experimental groups contained both male and female mice > 8 weeks of age. Since the conditioned fear response is not significant different between untreated males and females (see the Source data file), the sex was not considered in the study design and analysis. TH-IRES-Cre+/− mouse line was obtained from Jackson Laboratory (Bar Harbor, ME, USA) and maintained by backcrossing to C57/B6J mice. The study included Th-Cre mice (four males and two females) injected with AAV5-synP-DIO-eGFP-WPRE-hGH virus, seven C57BL/6J mice (four females and three males) for the FFN270 experiment, twenty-four mice C57BL/6J mice (eighteen females and six males) injected with AAV9-GRABNE, fifteen Th-Cre mice injected with DREADD hM4D(Gi)-mCherry (seven females and eight males) vs 11 Th-Cre mice (three females and eight males) injected with the control YFP virus for the chemogenetic inhibition of LC axons in the CB during conditioning, ten Th-Cre mice (six females and four males) injected with Arch3-YFP virus vs 3 Th-Cre mice (two females and one male) injected with the control YFP virus for the optogenetic inhibition of LC axons in the CB during conditioning, seven Th-Cre mice injected with Arch3-YFP virus for the optogenetic inhibition of LC axons in the CB during recall.

Field-collected samples

No data was collected from the field in this study.

Ethics oversight

All experimental procedures were approved by the Columbia University Institutional Animal Care and Use Committee (IACUC).

Note that full information on the approval of the study protocol must also be provided in the manuscript.
